# Crystal structures of two hydrogen-bonded compounds of chloranilic acid–ethyl­eneurea (1/1) and chloranilic acid–hydantoin (1/2)

**DOI:** 10.1107/S205698901801561X

**Published:** 2018-11-09

**Authors:** Kazuma Gotoh, Hiroyuki Ishida

**Affiliations:** a Department of Chemistry, Faculty of Science, Okayama University, Okayama 700-8530, Japan

**Keywords:** crystal structure, chloranilic acid, ethyleneurea, imidazolidin-2-one, hydantoin, imidazolidine-2,4-dione, hydrogen bond

## Abstract

The structures of the hydrogen-bonded 1:1 co-crystal of chloranilic acid (systematic name: 2,5-di­chloro-3,6-dihy­droxy-1,4-benzo­quinone) with ethyl­eneurea (systematic name: imidazolidin-2-one), C_6_H_2_Cl_2_O_4_·C_3_H_6_N_2_O, (I), and the 1:2 co-crystal of chloranilic acid with hydantoin (systematic name: imidazolidine-2,4-dione), C_6_H_2_Cl_2_O_4_·2C_3_H_4_N_2_O_2_, (II), have been determined at 180 K. In the crystals of both compounds, the base mol­ecules are in the lactam form and no acid–base inter­action involving H-atom transfer is observed. The asymmetric unit of (I) consists of two independent half-mol­ecules of chloranilic acid, with each of the acid mol­ecules lying about an inversion centre, and one ethyl­eneurea mol­ecule. The asymmetric unit of (II) consists of one half-mol­ecule of chloranilic acid, which lies about an inversion centre, and one hydantoin mol­ecule. In the crystal of (I), the acid and base mol­ecules are linked *via* O—H⋯O and N—H⋯O hydrogen bonds, forming an undulating sheet structure parallel to the *ab* plane. In (II), the base mol­ecules form an inversion dimer *via* a pair of N—H⋯O hydrogen bonds, and the base dimers are further linked through another N—H⋯O hydrogen bond into a layer structure parallel to (01). The acid mol­ecule and the base mol­ecule are linked *via* an O—H⋯O hydrogen bond.

## Abstract 2

The structures of the hydrogen-bonded 1:1 co-crystal of chloranilic acid with ethyl­eneurea and the 1:2 co-crystal of chloranilic acid with hydantoin have been determined at 180 K. In the crystals of both compounds, the base mol­ecules are in the lactam form and no acid–base inter­action involving H-atom transfer is observed. The acid and base mol­ecules are linked by short O—H⋯O and N—H⋯O hydrogen bonds.

## Chemical context   

Chloranilic acid, a dibasic acid with hydrogen-bond donor as well as acceptor groups, appears particularly attractive as a template for generating tightly bound self-assemblies with various organic bases, and also as a model compound for investigating hydrogen-transfer motions in O—H⋯N and N—H⋯O hydrogen-bonded systems (Zaman *et al.*, 2004 ▸; Seliger *et al.*, 2009 ▸; Asaji *et al.* 2010 ▸; Molčanov & Kojić-Prodić, 2010 ▸). In the present study, we have prepared two hydrogen-bonded compounds of chloranilic acid–ethyl­eneurea (1/1) and chloranilic acid–hydantoin (1/2) in order to extend our study on *D*—H⋯*A* hydrogen bonding (*D* = N, O, or C; *A* = N, O or Cl) in chloranilic acid–organic base systems (Gotoh & Ishida, 2017*a* ▸,*b* ▸, and references therein).
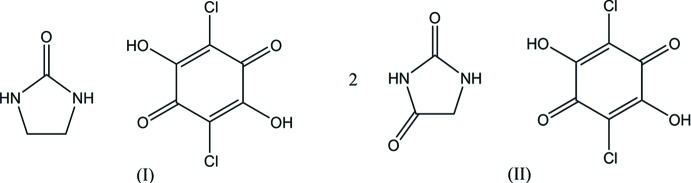


## Structural commentary   

**Figure 1:**
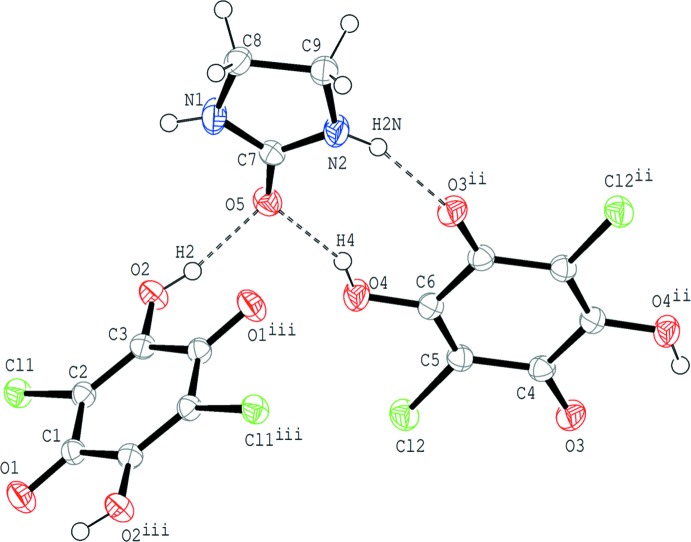
The mol­ecular structure of compound (I), showing the atom-numbering scheme. Displacement ellipsoids of non-H atoms are drawn at the 50% probability level and H atoms are drawn as small spheres of arbitrary radii. O—H⋯O and N—H⋯O hydrogen bonds are shown by dashed lines. [Symmetry codes: (ii) −*x*, −*y* + 1, −*z* + 1; (iii) −*x* + 1, −*y*, −*z* + 1.]

In compound (I), the base mol­ecule is in the lactam form and no acid–base inter­action involving H-atom transfer is observed (Fig. 1 ▸). In the asymmetric unit, there is one ethyl­eneurea mol­ecule and two crystallographically independent half-mol­ecules of chloranilic acid, with each of the acid mol­ecules lying about an inversion centre. The O atom of ethyl­eneurea participates in two O—H⋯O hydrogen bonds as an acceptor for two O—H groups of chloranilic acid (O2—H2⋯O5 and O4—H4⋯O5; Table 1 ▸). The base ring (C7/N1/C8/C9/N2) is essentially planar and makes dihedral angles of 88.75 (6) and 3.27 (6)°, respectively, with the acid C1–C3/C1^iii^–C3^iii^ and C4–C6/C4^ii^–C6^ii^ rings [symmetry codes: (ii) −*x*, −*y* + 1, −*z* + 1; (iii) −*x* + 1, −*y*, −*z* + 1].

**Figure 2:**
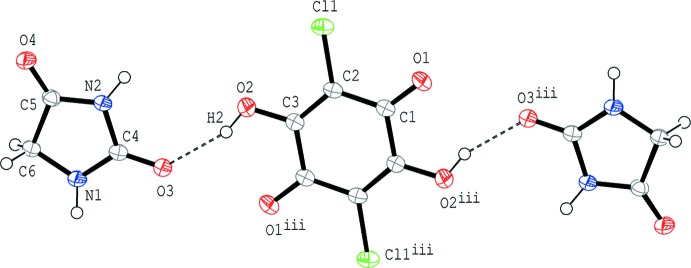
The mol­ecular structure of compound (II), showing the atom-numbering scheme. Displacement ellipsoids of non-H atoms are drawn at the 50% probability level and H atoms are drawn as small spheres of arbitrary radii. O—H⋯O hydrogen bonds are shown by dashed lines. [Symmetry code: (iii) −*x* + 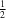, −*y* + 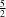, −*z* + 1.]

In compound (II), the base mol­ecule is also in the lactam form and no acid–base inter­action involving H-atom transfer is observed (Fig. 2 ▸). The chloranilic acid mol­ecule is located on an inversion centre and the asymmetric unit consists of one hydantoin mol­ecule and a half-mol­ecule of chloranilic acid. The acid and base mol­ecules are linked *via* an O—H⋯O hydrogen bond (O2—H2⋯O3; Table 2 ▸), forming a centrosymmetric 1:2 aggregate of the acid and the base. The 1:2 unit is approximately planar with a dihedral angle of 5.42 (5)° between the acid and base rings.

## Supra­molecular features   

**Figure 3:**
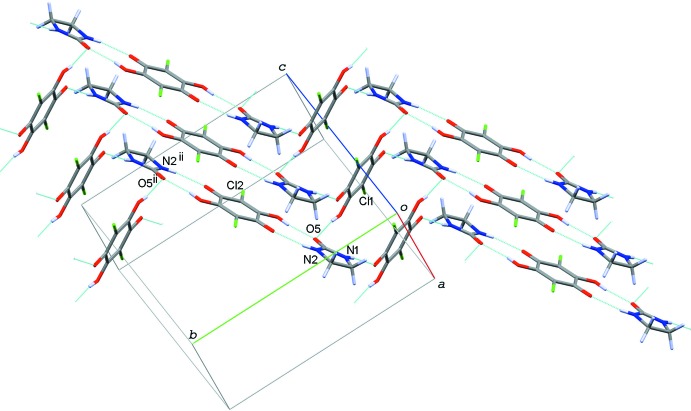
A partial packing diagram of compound (I), showing the undulating sheet structure formed by O—H⋯O and N—H⋯O hydrogen bonds (light-blue dotted lines). [Symmetry code: (ii) −*x*, −*y* + 1, −*z* + 1.]

In the crystal of compound (I), the acid and base mol­ecules are alternately arranged through O—H⋯O and N—H⋯O hydrogen bonds (O4—H4⋯O5, N1—H1*N*⋯O1^i^, N2—H2*H*⋯O3^ii^; symmetry codes as in Table 1 ▸), forming an undulating tape structure along [3
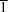
0]. The tapes are stacked along the *a* axis *via* another O—H⋯O hydrogen bond (O2—H2—O5; Table 1 ▸) into a sheet structure parallel to the *ab* plane (Fig. 3 ▸).

**Figure 4:**
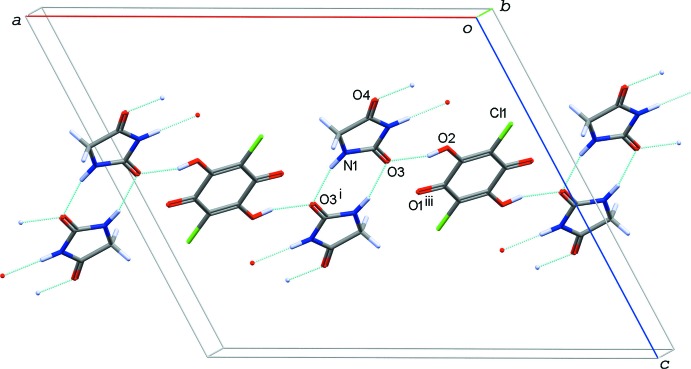
A partial packing diagram of compound (II) viewed approximately along the *b* axis, showing a hydrogen-bonded tape structure formed by acid mol­ecules and pairs of base mol­ecules. O—H⋯O and N—H⋯O hydrogen bonds are shown by light-blue dotted lines. [Symmetry codes: (i) −*x* + 1, −*y* + 1, −*z* + 1; (iii) −*x* + 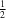, −*y* + 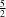, −*z* + 1.]

**Figure 5:**
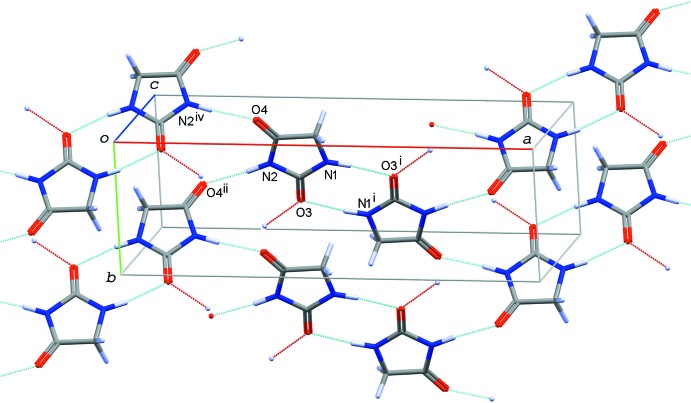
A partial packing diagram of compound (II), showing hydrogen-bonding scheme in the layer formed by base mol­ecules. N—H⋯O hydrogen bonds between the base mol­ecules are shown by light-blue dotted lines, while O—H⋯O hydrogen bonds between the base and acid mol­ecules are shown by red dotted lines. [Symmetry codes: (i) −*x* + 1, −*y* + 1, −*z* + 1; (ii) −*x* + 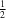, *y* + 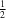, −*z* + 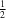; (iv) −*x* + 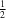, *y* − 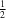, −*z* + 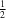.]

In the crystal of (II), two adjacent base mol­ecules, which are related by an inversion centre, form a dimer *via* a pair of N—H⋯O hydrogen bonds (N1—H1*N*⋯O3^i^; symmetry code as in Table 2 ▸), and the base dimer and the acid mol­ecule are alternately linked through an O—H⋯O hydrogen bond (O2—H2⋯O3; Table 2 ▸), forming a flat tape structure along the *a-*axis direction (Fig. 4 ▸). The base dimers are assembled *via* another N—H⋯O hydrogen bond (N2—H2*N*⋯O4^ii^; symmetry code as in Table 2 ▸), forming a layer parallel to (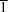
01) as shown in Fig. 5 ▸. The O—H⋯O hydrogen bond (O2—H2⋯O3; Table 2 ▸) formed between the acid and base mol­ecules links the layers.

## Database survey   

A search of the Cambridge Structural Database (Version 5.39, last update August 2018; Groom *et al.*, 2016 ▸) for organic crystals of chloranilic acid with lactam-form base mol­ecules gave ten hits. In the seven crystals of these compounds, O—H⋯O hydrogen bonds between the O—H group of chloranilic acid and the carbonyl group of base are observed [refcodes ACOJIO (Gotoh & Ishida, 2017*a* ▸), AJAGIB (Luo & Palmore, 2002 ▸), HUFZUE (Jasinski *et al.*, 2010 ▸), ODIHIU, SADTIC, SADTOI and SADTUO (Gotoh & Ishida, 2011 ▸)]. In particular, the compounds of chloranilic acid with 2-pyridone (ACOJIO), gabapentin-lactum (HUFZUE), pyrrolidin-2-one (ODIHIU) and piperidin-2-one (SADTUO) show short O—H⋯O hydrogen bonds (O⋯O shorter than 2.5 Å). In the O—H⋯O hydrogen bond [O⋯O = 2.4484 (10) Å] of chloranilic acid–piperidin-2-one (1/2) (SADTUO), the H atom is disordered over two positions.

## Synthesis and crystallization   

Single crystals of compound (I) were obtained by slow evaporation from an aceto­nitrile solution (150 ml) of chloranilic acid (330 mg) with ethyl­eneurea (140 mg) at room temperature. Crystals of compound (II) were obtained by slow evaporation from an aceto­nitrile solution (250 ml) of chloranilic acid (350 mg) with hydantoin (340 mg) at room temperature.

## Refinement   

Crystal data, data collection and structure refinement details are summarized in Table 3 ▸. All H atoms in compounds (I) and (II) were found in difference Fourier maps. The O- and N-bound H atoms were freely refined. C-bound H atoms were positioned geometrically (C—H = 0.99 Å) and were treated as riding with *U*_iso_(H) = 1.2*U*_eq_(C).

## Supplementary Material

Crystal structure: contains datablock(s) General, I, II. DOI: 10.1107/S205698901801561X/lh5884sup1.cif

Structure factors: contains datablock(s) I. DOI: 10.1107/S205698901801561X/lh5884Isup2.hkl

Structure factors: contains datablock(s) II. DOI: 10.1107/S205698901801561X/lh5884IIsup3.hkl

Supporting information file. DOI: 10.1107/S205698901801561X/lh5884Isup4.cml

Supporting information file. DOI: 10.1107/S205698901801561X/lh5884IIsup5.cml

CCDC references: 1876998, 1876997

Additional supporting information:  crystallographic information; 3D view; checkCIF report

## Figures and Tables

**Table 1:** Hydrogen-bond geometry (Å, °) for (I)

*D*—H⋯*A*	*D*—H	H⋯*A*	*D*⋯*A*	*D*—H⋯*A*
O2—H2⋯O5	0.81 (2)	1.82 (2)	2.6090 (11)	164 (2)
O4—H4⋯O5	0.86 (2)	1.88 (2)	2.6635 (12)	151.0 (19)
N1—H1*N*⋯O1^i^	0.85 (2)	2.06 (2)	2.9003 (15)	168 (2)
N2—H2*N*⋯O3^ii^	0.85 (2)	2.06 (2)	2.8654 (15)	158.6 (17)

Symmetry codes: (i) 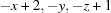; (ii) 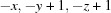.

**Table 2:** Hydrogen-bond geometry (Å, °) for (II)

*D*—H⋯*A*	*D*—H	H⋯*A*	*D*⋯*A*	*D*—H⋯*A*
O2—H2⋯O3	0.86 (2)	1.97 (2)	2.7917 (15)	160 (2)
N1—H1*N*⋯O3^i^	0.91 (2)	2.00 (2)	2.8927 (13)	165 (2)
N2—H2*N*⋯O4^ii^	0.91 (2)	1.85 (2)	2.7560 (14)	176 (2)

Symmetry codes: (i) 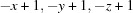; (ii) 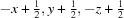.

**Table 3:** Experimental details

	(I)	(II)
Crystal data		
Chemical formula	C_6_H_2_Cl_2_O_4_·C_3_H_6_N_2_O	C_6_H_2_Cl_2_O_4_·2C_3_H_4_N_2_O_2_
*M*_r_	295.08	409.14
Crystal system, space group	Monoclinic, *P*2_1_/*c*	Monoclinic, *C*2/*c*
Temperature (K)	180	180
*a*, *b*, *c* (Å)	5.0180 (4), 14.6142 (10), 15.8882 (11)	19.5690 (8), 5.18661 (10), 16.6103 (3)
β (°)	105.563 (3)	117.965 (3)
*V* (Å^3^)	1122.43 (15)	1489.03 (8)
*Z*	4	4
Radiation type	Mo *K*α	Mo *K*α
μ (mm^−1^)	0.59	0.49
Crystal size (mm)	0.45 × 0.29 × 0.23	0.49 × 0.33 × 0.24

Data collection		
Diffractometer	Rigaku R-AXIS RAPIDII	Rigaku R-AXIS RAPIDII
Absorption correction	Numerical (*NUMABS*; Higashi, 1999 ▸)	Numerical (*NUMABS*; Higashi, 1999 ▸)
*T*_min_, *T*_max_	0.716, 0.873	0.808, 0.888
No. of measured, independent and observed [*I* > 2σ(*I*)] reflections	21546, 3266, 3040	14622, 2181, 2029
*R*_int_	0.057	0.072
(sin θ/λ)_max_ (Å^−1^)	0.704	0.704

Refinement		
*R*[*F* ^2^ > 2σ(*F* ^2^)], *wR*(*F* ^2^), *S*	0.033, 0.092, 1.07	0.036, 0.100, 1.08
No. of reflections	3266	2181
No. of parameters	179	130
H-atom treatment	H atoms treated by a mixture of independent and constrained refinement	H atoms treated by a mixture of independent and constrained refinement
Δρ_max_, Δρ_min_ (e Å^−3^)	0.54, −0.31	0.44, −0.40

Computer programs: *RAPID-AUTO* (Rigaku, 2006 ▸), *SIR92* (Altomare *et al.*, 1993 ▸), *SHELXT2018* (Sheldrick, 2015*a* ▸), *SHELXL2018* (Sheldrick, 2015*b* ▸), *ORTEP-3* for Windows (Farrugia, 2012 ▸), *Mercury* (Macrae *et al.*, 2006 ▸), *CrystalStructure* (Rigaku, 2018 ▸) and *PLATON* (Spek, 2015 ▸).

## supplementary crystallographic information

### 2,5-Dichloro-3,6-dihydroxy-1,4-benzoquinone–imidazolidin-2-one (1/1) (I) Crystal data

**Table:**

C_6_H_2_Cl_2_O_4_·C_3_H_6_N_2_O	*F*(000) = 600.00
*M**_r_* = 295.08	*D*_x_ = 1.746 Mg m^−^^3^
Monoclinic, *P*2_1_/*c*	Mo *K*α radiation, λ = 0.71075 Å
*a* = 5.0180 (4) Å	Cell parameters from 19204 reflections
*b* = 14.6142 (10) Å	θ = 3.0–30.1°
*c* = 15.8882 (11) Å	µ = 0.59 mm^−^^1^
β = 105.563 (3)°	*T* = 180 K
*V* = 1122.43 (15) Å^3^	Block, brown
*Z* = 4	0.45 × 0.29 × 0.23 mm

### 2,5-Dichloro-3,6-dihydroxy-1,4-benzoquinone–imidazolidin-2-one (1/1) (I) Data collection

**Table:**

Rigaku R-AXIS RAPIDII diffractometer	3040 reflections with *I* > 2σ(*I*)
Detector resolution: 10.000 pixels mm^-1^	*R*_int_ = 0.057
ω scans	θ_max_ = 30.0°, θ_min_ = 3.0°
Absorption correction: numerical (NUMABS; Higashi, 1999)	*h* = −7→7
*T*_min_ = 0.716, *T*_max_ = 0.873	*k* = −20→19
21546 measured reflections	*l* = −21→22
3266 independent reflections	

### 2,5-Dichloro-3,6-dihydroxy-1,4-benzoquinone–imidazolidin-2-one (1/1) (I) Refinement

**Table:**

Refinement on *F*^2^	Secondary atom site location: difference Fourier map
*R*[*F*^2^ > 2σ(*F*^2^)] = 0.033	Hydrogen site location: mixed
*wR*(*F*^2^) = 0.092	H atoms treated by a mixture of independent and constrained refinement
*S* = 1.07	*w* = 1/[σ^2^(*F*_o_^2^) + (0.0573*P*)^2^ + 0.2007*P*] where *P* = (*F*_o_^2^ + 2*F*_c_^2^)/3
3266 reflections	(Δ/σ)_max_ = 0.001
179 parameters	Δρ_max_ = 0.54 e Å^−^^3^
0 restraints	Δρ_min_ = −0.31 e Å^−^^3^
Primary atom site location: structure-invariant direct methods	

### 2,5-Dichloro-3,6-dihydroxy-1,4-benzoquinone–imidazolidin-2-one (1/1) (I) Special details

**Table:**

Geometry. All esds (except the esd in the dihedral angle between two l.s. planes) are estimated using the full covariance matrix. The cell esds are taken into account individually in the estimation of esds in distances, angles and torsion angles; correlations between esds in cell parameters are only used when they are defined by crystal symmetry. An approximate (isotropic) treatment of cell esds is used for estimating esds involving l.s. planes.

### 2,5-Dichloro-3,6-dihydroxy-1,4-benzoquinone–imidazolidin-2-one (1/1) (I) Fractional atomic coordinates and isotropic or equivalent isotropic displacement parameters (Å^2^)

**Table:**

	*x*	*y*	*z*	*U*_iso_*/*U*_eq_	
Cl1	1.06653 (5)	0.00389 (2)	0.64704 (2)	0.02517 (9)	
Cl2	0.28647 (6)	0.43040 (2)	0.69108 (2)	0.03101 (9)	
O1	0.64834 (18)	−0.14266 (6)	0.60507 (6)	0.03198 (19)	
O2	0.83758 (17)	0.15225 (5)	0.51887 (5)	0.02612 (17)	
O3	−0.17821 (19)	0.55990 (6)	0.63245 (6)	0.03138 (19)	
O4	0.43441 (18)	0.37722 (6)	0.52805 (6)	0.03039 (19)	
O5	0.70481 (17)	0.29928 (5)	0.42370 (5)	0.02644 (17)	
N1	0.8521 (2)	0.24152 (9)	0.30842 (7)	0.0372 (3)	
N2	0.4933 (2)	0.33134 (8)	0.27881 (7)	0.0323 (2)	
C1	0.5873 (2)	−0.07583 (7)	0.55745 (7)	0.02118 (19)	
C2	0.7619 (2)	0.00483 (7)	0.56565 (7)	0.02058 (19)	
C3	0.6846 (2)	0.07805 (7)	0.51226 (6)	0.02064 (19)	
C4	−0.0902 (2)	0.53136 (7)	0.57314 (7)	0.0233 (2)	
C5	0.1390 (2)	0.46653 (7)	0.58624 (7)	0.0238 (2)	
C6	0.2263 (2)	0.43578 (7)	0.51801 (7)	0.0236 (2)	
C7	0.6858 (2)	0.29121 (7)	0.34329 (7)	0.0224 (2)	
C8	0.7865 (2)	0.24974 (8)	0.21442 (8)	0.0275 (2)	
H8A	0.737465	0.189643	0.185683	0.033*	
H8B	0.943169	0.276222	0.195803	0.033*	
C9	0.5369 (3)	0.31466 (9)	0.19376 (8)	0.0325 (2)	
H9A	0.578932	0.372186	0.166986	0.039*	
H9B	0.372898	0.285493	0.153938	0.039*	
H1N	0.989 (4)	0.2121 (14)	0.3405 (14)	0.059 (6)*	
H2N	0.381 (4)	0.3687 (13)	0.2915 (12)	0.041 (5)*	
H2	0.766 (4)	0.1933 (16)	0.4861 (14)	0.056 (6)*	
H4	0.476 (4)	0.3613 (13)	0.4811 (13)	0.045 (5)*	

### 2,5-Dichloro-3,6-dihydroxy-1,4-benzoquinone–imidazolidin-2-one (1/1) (I) Atomic displacement parameters (Å^2^)

**Table:**

	*U*^11^	*U*^22^	*U*^33^	*U*^12^	*U*^13^	*U*^23^
Cl1	0.02251 (14)	0.02897 (15)	0.02081 (14)	−0.00049 (8)	0.00027 (10)	0.00183 (8)
Cl2	0.03685 (16)	0.03457 (16)	0.02165 (15)	0.00812 (10)	0.00789 (11)	0.00275 (9)
O1	0.0284 (4)	0.0277 (4)	0.0345 (4)	−0.0009 (3)	−0.0007 (3)	0.0114 (3)
O2	0.0273 (4)	0.0226 (4)	0.0252 (4)	−0.0031 (3)	0.0014 (3)	0.0039 (3)
O3	0.0355 (4)	0.0356 (4)	0.0262 (4)	0.0082 (3)	0.0138 (3)	−0.0009 (3)
O4	0.0333 (4)	0.0350 (4)	0.0243 (4)	0.0124 (3)	0.0100 (3)	0.0016 (3)
O5	0.0319 (4)	0.0244 (4)	0.0230 (4)	0.0017 (3)	0.0071 (3)	0.0006 (3)
N1	0.0378 (6)	0.0468 (6)	0.0272 (5)	0.0224 (5)	0.0093 (4)	0.0057 (4)
N2	0.0336 (5)	0.0411 (5)	0.0240 (5)	0.0163 (4)	0.0106 (4)	0.0045 (4)
C1	0.0207 (4)	0.0220 (4)	0.0204 (4)	0.0018 (3)	0.0047 (3)	0.0012 (3)
C2	0.0192 (4)	0.0236 (5)	0.0178 (4)	0.0012 (3)	0.0029 (3)	0.0002 (3)
C3	0.0215 (4)	0.0221 (4)	0.0181 (4)	0.0003 (3)	0.0049 (3)	−0.0006 (3)
C4	0.0253 (5)	0.0233 (5)	0.0225 (5)	−0.0001 (4)	0.0083 (4)	−0.0004 (4)
C5	0.0260 (5)	0.0245 (5)	0.0213 (5)	0.0019 (4)	0.0070 (4)	0.0010 (4)
C6	0.0244 (5)	0.0231 (5)	0.0240 (5)	0.0016 (3)	0.0077 (4)	0.0002 (3)
C7	0.0239 (4)	0.0191 (4)	0.0246 (5)	−0.0005 (3)	0.0070 (4)	0.0021 (3)
C8	0.0254 (5)	0.0315 (5)	0.0275 (5)	0.0034 (4)	0.0100 (4)	−0.0007 (4)
C9	0.0346 (6)	0.0416 (6)	0.0240 (5)	0.0125 (5)	0.0127 (4)	0.0075 (4)

### 2,5-Dichloro-3,6-dihydroxy-1,4-benzoquinone–imidazolidin-2-one (1/1) (I) Geometric parameters (Å, º)

**Table:**

Cl1—C2	1.7169 (10)	N2—C9	1.4464 (15)
Cl2—C5	1.7145 (11)	N2—H2N	0.849 (19)
O1—C1	1.2230 (13)	C1—C2	1.4537 (14)
O2—C3	1.3165 (12)	C1—C3^i^	1.5092 (14)
O2—H2	0.81 (2)	C2—C3	1.3560 (14)
O3—C4	1.2168 (13)	C4—C5	1.4612 (14)
O4—C6	1.3264 (12)	C4—C6^ii^	1.5045 (15)
O4—H4	0.86 (2)	C5—C6	1.3504 (15)
O5—C7	1.2609 (13)	C8—C9	1.5348 (15)
N1—C7	1.3338 (14)	C8—H8A	0.9900
N1—C8	1.4455 (15)	C8—H8B	0.9900
N1—H1N	0.85 (2)	C9—H9A	0.9900
N2—C7	1.3397 (14)	C9—H9B	0.9900
			
C3—O2—H2	114.1 (14)	C6—C5—Cl2	121.94 (8)
C6—O4—H4	115.8 (13)	C4—C5—Cl2	117.13 (8)
C7—N1—C8	112.92 (10)	O4—C6—C5	122.18 (10)
C7—N1—H1N	121.2 (15)	O4—C6—C4^ii^	117.46 (9)
C8—N1—H1N	125.7 (15)	C5—C6—C4^ii^	120.35 (9)
C7—N2—C9	112.46 (10)	O5—C7—N1	125.87 (10)
C7—N2—H2N	119.3 (12)	O5—C7—N2	125.24 (10)
C9—N2—H2N	127.5 (12)	N1—C7—N2	108.88 (10)
O1—C1—C2	123.17 (9)	N1—C8—C9	102.65 (9)
O1—C1—C3^i^	117.64 (9)	N1—C8—H8A	111.2
C2—C1—C3^i^	119.19 (8)	C9—C8—H8A	111.2
C3—C2—C1	121.28 (9)	N1—C8—H8B	111.2
C3—C2—Cl1	121.68 (8)	C9—C8—H8B	111.2
C1—C2—Cl1	117.03 (7)	H8A—C8—H8B	109.2
O2—C3—C2	122.48 (9)	N2—C9—C8	102.92 (9)
O2—C3—C1^i^	117.99 (9)	N2—C9—H9A	111.2
C2—C3—C1^i^	119.53 (9)	C8—C9—H9A	111.2
O3—C4—C5	123.19 (10)	N2—C9—H9B	111.2
O3—C4—C6^ii^	118.08 (10)	C8—C9—H9B	111.2
C5—C4—C6^ii^	118.73 (9)	H9A—C9—H9B	109.1
C6—C5—C4	120.92 (9)		
			
O1—C1—C2—C3	−179.04 (11)	C4—C5—C6—O4	179.73 (10)
C3^i^—C1—C2—C3	0.34 (16)	Cl2—C5—C6—O4	−1.31 (16)
O1—C1—C2—Cl1	−0.19 (14)	C4—C5—C6—C4^ii^	0.61 (17)
C3^i^—C1—C2—Cl1	179.19 (7)	Cl2—C5—C6—C4^ii^	179.57 (8)
C1—C2—C3—O2	179.32 (9)	C8—N1—C7—O5	−176.92 (10)
Cl1—C2—C3—O2	0.52 (15)	C8—N1—C7—N2	3.23 (15)
C1—C2—C3—C1^i^	−0.34 (16)	C9—N2—C7—O5	175.71 (11)
Cl1—C2—C3—C1^i^	−179.14 (7)	C9—N2—C7—N1	−4.43 (15)
O3—C4—C5—C6	178.94 (11)	C7—N1—C8—C9	−0.84 (14)
C6^ii^—C4—C5—C6	−0.60 (17)	C7—N2—C9—C8	3.73 (14)
O3—C4—C5—Cl2	−0.07 (15)	N1—C8—C9—N2	−1.64 (13)
C6^ii^—C4—C5—Cl2	−179.61 (8)		

Symmetry codes: (i) −*x*+1, −*y*, −*z*+1; (ii) −*x*, −*y*+1, −*z*+1.

### 2,5-Dichloro-3,6-dihydroxy-1,4-benzoquinone–imidazolidin-2-one (1/1) (I) Hydrogen-bond geometry (Å, º)

**Table:**

*D*—H···*A*	*D*—H	H···*A*	*D*···*A*	*D*—H···*A*
O2—H2···O5	0.81 (2)	1.82 (2)	2.6090 (11)	164 (2)
O4—H4···O5	0.86 (2)	1.88 (2)	2.6635 (12)	151.0 (19)
N1—H1*N*···O1^iii^	0.85 (2)	2.06 (2)	2.9003 (15)	168 (2)
N2—H2*N*···O3^ii^	0.85 (2)	2.06 (2)	2.8654 (15)	158.6 (17)

Symmetry codes: (ii) −*x*, −*y*+1, −*z*+1; (iii) −*x*+2, −*y*, −*z*+1.

### 2,5-Dichloro-3,6-dihydroxy-1,4-benzoquinone–imidazolidine-2,4-dione (1/2) (II) Crystal data

**Table:**

C_6_H_2_Cl_2_O_4_·2C_3_H_4_N_2_O_2_	*F*(000) = 832.00
*M**_r_* = 409.14	*D*_x_ = 1.825 Mg m^−^^3^
Monoclinic, *C*2/*c*	Mo *K*α radiation, λ = 0.71075 Å
*a* = 19.5690 (8) Å	Cell parameters from 13670 reflections
*b* = 5.18661 (10) Å	θ = 3.3–30.2°
*c* = 16.6103 (3) Å	µ = 0.49 mm^−^^1^
β = 117.965 (3)°	*T* = 180 K
*V* = 1489.03 (8) Å^3^	Block, brown
*Z* = 4	0.49 × 0.33 × 0.24 mm

### 2,5-Dichloro-3,6-dihydroxy-1,4-benzoquinone–imidazolidine-2,4-dione (1/2) (II) Data collection

**Table:**

Rigaku R-AXIS RAPIDII diffractometer	2029 reflections with *I* > 2σ(*I*)
Detector resolution: 10.000 pixels mm^-1^	*R*_int_ = 0.072
ω scans	θ_max_ = 30.0°, θ_min_ = 4.1°
Absorption correction: numerical (NUMABS; Higashi, 1999)	*h* = −27→27
*T*_min_ = 0.808, *T*_max_ = 0.888	*k* = −7→7
14622 measured reflections	*l* = −22→23
2181 independent reflections	

### 2,5-Dichloro-3,6-dihydroxy-1,4-benzoquinone–imidazolidine-2,4-dione (1/2) (II) Refinement

**Table:**

Refinement on *F*^2^	Secondary atom site location: difference Fourier map
*R*[*F*^2^ > 2σ(*F*^2^)] = 0.036	Hydrogen site location: mixed
*wR*(*F*^2^) = 0.100	H atoms treated by a mixture of independent and constrained refinement
*S* = 1.08	*w* = 1/[σ^2^(*F*_o_^2^) + (0.0633*P*)^2^ + 0.4572*P*] where *P* = (*F*_o_^2^ + 2*F*_c_^2^)/3
2181 reflections	(Δ/σ)_max_ < 0.001
130 parameters	Δρ_max_ = 0.44 e Å^−^^3^
0 restraints	Δρ_min_ = −0.40 e Å^−^^3^
Primary atom site location: structure-invariant direct methods	

### 2,5-Dichloro-3,6-dihydroxy-1,4-benzoquinone–imidazolidine-2,4-dione (1/2) (II) Special details

**Table:**

Geometry. All esds (except the esd in the dihedral angle between two l.s. planes) are estimated using the full covariance matrix. The cell esds are taken into account individually in the estimation of esds in distances, angles and torsion angles; correlations between esds in cell parameters are only used when they are defined by crystal symmetry. An approximate (isotropic) treatment of cell esds is used for estimating esds involving l.s. planes.
Refinement. Refinement was performed using all reflections. The weighted R-factor (wR) and goodness of fit (S) are based on F^2^. R-factor (gt) are based on F. The threshold expression of F^2^ > 2.0 sigma(F^2^) is used only for calculating R-factor (gt).

### 2,5-Dichloro-3,6-dihydroxy-1,4-benzoquinone–imidazolidine-2,4-dione (1/2) (II) Fractional atomic coordinates and isotropic or equivalent isotropic displacement parameters (Å^2^)

**Table:**

	*x*	*y*	*z*	*U*_iso_*/*U*_eq_	
Cl1	0.09683 (2)	1.02553 (6)	0.33978 (2)	0.02980 (12)	
O1	0.11596 (5)	1.47273 (17)	0.45960 (6)	0.0313 (2)	
O2	0.25729 (5)	0.82514 (16)	0.40485 (6)	0.02794 (19)	
O3	0.39430 (5)	0.57413 (17)	0.43836 (6)	0.02824 (19)	
O4	0.31705 (4)	−0.10296 (18)	0.23989 (6)	0.0296 (2)	
N1	0.46359 (5)	0.26895 (19)	0.40528 (6)	0.0269 (2)	
N2	0.33612 (5)	0.25929 (18)	0.32811 (6)	0.02336 (19)	
C1	0.17655 (6)	1.3650 (2)	0.47556 (7)	0.0229 (2)	
C2	0.18138 (6)	1.1410 (2)	0.42588 (7)	0.0228 (2)	
C3	0.25018 (6)	1.0302 (2)	0.44779 (7)	0.0223 (2)	
C4	0.39913 (6)	0.3869 (2)	0.39566 (7)	0.0226 (2)	
C5	0.35871 (6)	0.0519 (2)	0.29613 (7)	0.0229 (2)	
C6	0.44610 (6)	0.0494 (2)	0.34539 (8)	0.0255 (2)	
H6A	0.466307	−0.112237	0.380266	0.031*	
H6B	0.467588	0.070964	0.302420	0.031*	
H1N	0.5127 (12)	0.308 (4)	0.4484 (15)	0.054 (5)*	
H2	0.3043 (12)	0.772 (4)	0.4269 (14)	0.049 (5)*	
H2N	0.2861 (11)	0.313 (4)	0.3055 (13)	0.045 (5)*	

### 2,5-Dichloro-3,6-dihydroxy-1,4-benzoquinone–imidazolidine-2,4-dione (1/2) (II) Atomic displacement parameters (Å^2^)

**Table:**

	*U*^11^	*U*^22^	*U*^33^	*U*^12^	*U*^13^	*U*^23^
Cl1	0.02000 (16)	0.03480 (18)	0.02881 (17)	−0.00423 (8)	0.00662 (12)	−0.00241 (9)
O1	0.0206 (4)	0.0327 (4)	0.0358 (4)	0.0052 (3)	0.0091 (3)	−0.0012 (3)
O2	0.0237 (4)	0.0269 (4)	0.0314 (4)	0.0015 (3)	0.0114 (3)	−0.0036 (3)
O3	0.0219 (4)	0.0300 (4)	0.0288 (4)	0.0015 (3)	0.0085 (3)	−0.0058 (3)
O4	0.0198 (4)	0.0324 (4)	0.0321 (4)	−0.0038 (3)	0.0085 (3)	−0.0086 (3)
N1	0.0160 (4)	0.0312 (5)	0.0288 (4)	−0.0018 (3)	0.0067 (3)	−0.0071 (4)
N2	0.0158 (4)	0.0268 (4)	0.0239 (4)	0.0003 (3)	0.0063 (3)	−0.0025 (3)
C1	0.0191 (4)	0.0254 (5)	0.0226 (4)	0.0009 (3)	0.0084 (4)	0.0032 (4)
C2	0.0177 (4)	0.0257 (5)	0.0220 (4)	−0.0009 (3)	0.0069 (3)	0.0020 (4)
C3	0.0205 (4)	0.0232 (5)	0.0226 (4)	0.0002 (3)	0.0094 (4)	0.0021 (3)
C4	0.0180 (4)	0.0256 (5)	0.0221 (4)	−0.0010 (3)	0.0076 (3)	0.0003 (4)
C5	0.0173 (4)	0.0262 (5)	0.0239 (5)	−0.0002 (3)	0.0086 (4)	−0.0002 (4)
C6	0.0162 (4)	0.0276 (5)	0.0295 (5)	−0.0008 (3)	0.0079 (4)	−0.0049 (4)

### 2,5-Dichloro-3,6-dihydroxy-1,4-benzoquinone–imidazolidine-2,4-dione (1/2) (II) Geometric parameters (Å, º)

**Table:**

Cl1—C2	1.7094 (10)	N2—C5	1.3620 (14)
O1—C1	1.2222 (12)	N2—C4	1.3857 (13)
O2—C3	1.3237 (13)	N2—H2N	0.912 (18)
O2—H2	0.86 (2)	C1—C2	1.4536 (15)
O3—C4	1.2318 (14)	C1—C3^i^	1.5035 (14)
O4—C5	1.2117 (13)	C2—C3	1.3475 (14)
N1—C4	1.3425 (13)	C5—C6	1.5106 (14)
N1—C6	1.4436 (14)	C6—H6A	0.9900
N1—H1N	0.91 (2)	C6—H6B	0.9900
			
C3—O2—H2	112.8 (14)	O2—C3—C1^i^	116.58 (9)
C4—N1—C6	111.82 (8)	C2—C3—C1^i^	120.65 (9)
C4—N1—H1N	125.3 (13)	O3—C4—N1	127.75 (10)
C6—N1—H1N	122.5 (13)	O3—C4—N2	124.29 (9)
C5—N2—C4	111.40 (8)	N1—C4—N2	107.95 (9)
C5—N2—H2N	124.3 (12)	O4—C5—N2	126.87 (10)
C4—N2—H2N	124.2 (12)	O4—C5—C6	126.41 (10)
O1—C1—C2	123.86 (10)	N2—C5—C6	106.71 (9)
O1—C1—C3^i^	117.46 (10)	N1—C6—C5	102.06 (8)
C2—C1—C3^i^	118.67 (8)	N1—C6—H6A	111.4
C3—C2—C1	120.68 (9)	C5—C6—H6A	111.4
C3—C2—Cl1	121.94 (8)	N1—C6—H6B	111.4
C1—C2—Cl1	117.37 (7)	C5—C6—H6B	111.4
O2—C3—C2	122.77 (10)	H6A—C6—H6B	109.2
			
O1—C1—C2—C3	−179.33 (10)	C6—N1—C4—N2	−1.64 (12)
C3^i^—C1—C2—C3	−0.32 (16)	C5—N2—C4—O3	−176.84 (11)
O1—C1—C2—Cl1	0.48 (15)	C5—N2—C4—N1	2.61 (12)
C3^i^—C1—C2—Cl1	179.49 (7)	C4—N2—C5—O4	176.75 (11)
C1—C2—C3—O2	179.56 (9)	C4—N2—C5—C6	−2.45 (12)
Cl1—C2—C3—O2	−0.24 (15)	C4—N1—C6—C5	0.19 (12)
C1—C2—C3—C1^i^	0.32 (16)	O4—C5—C6—N1	−177.85 (11)
Cl1—C2—C3—C1^i^	−179.48 (7)	N2—C5—C6—N1	1.35 (11)
C6—N1—C4—O3	177.78 (11)		

Symmetry code: (i) −*x*+1/2, −*y*+5/2, −*z*+1.

### 2,5-Dichloro-3,6-dihydroxy-1,4-benzoquinone–imidazolidine-2,4-dione (1/2) (II) Hydrogen-bond geometry (Å, º)

**Table:**

*D*—H···*A*	*D*—H	H···*A*	*D*···*A*	*D*—H···*A*
O2—H2···O3	0.86 (2)	1.97 (2)	2.7917 (15)	160 (2)
N1—H1*N*···O3^ii^	0.91 (2)	2.00 (2)	2.8927 (13)	165 (2)
N2—H2*N*···O4^iii^	0.91 (2)	1.85 (2)	2.7560 (14)	176 (2)

Symmetry codes: (ii) −*x*+1, −*y*+1, −*z*+1; (iii) −*x*+1/2, *y*+1/2, −*z*+1/2.
